# Direct Laser Writing of Chitosan–Borax Composites: Toward Sustainable Electrochemical Sensors

**DOI:** 10.1021/acssuschemeng.3c02708

**Published:** 2023-09-01

**Authors:** Eoghan Vaughan, Chiara Santillo, Alessandra Imbrogno, Gennaro Gentile, Aidan J. Quinn, Saulius Kaciulis, Marino Lavorgna, Daniela Iacopino

**Affiliations:** † Tyndall National Institute, University College Cork, Lee Maltings Complex, Dyke Parade, Cork T12R5CP, Ireland; ‡ Institute for Polymers, Composites and Biomaterials, National Research Council of Italy, P.le E. Fermi 1, 80055 Portici, Italy; § Institute for Polymers Composites and Biomaterials, National Research Council of Italy, Via Campi Flegrei 34, 80078 Pozzuoli, Italy; ∥ Institute for the Study of Nanostructured Materials, National Research Council, Monterotondo Staz., 00015 Rome, Italy

**Keywords:** laser-induced graphene, chitosan, composite, borax, char formation

## Abstract

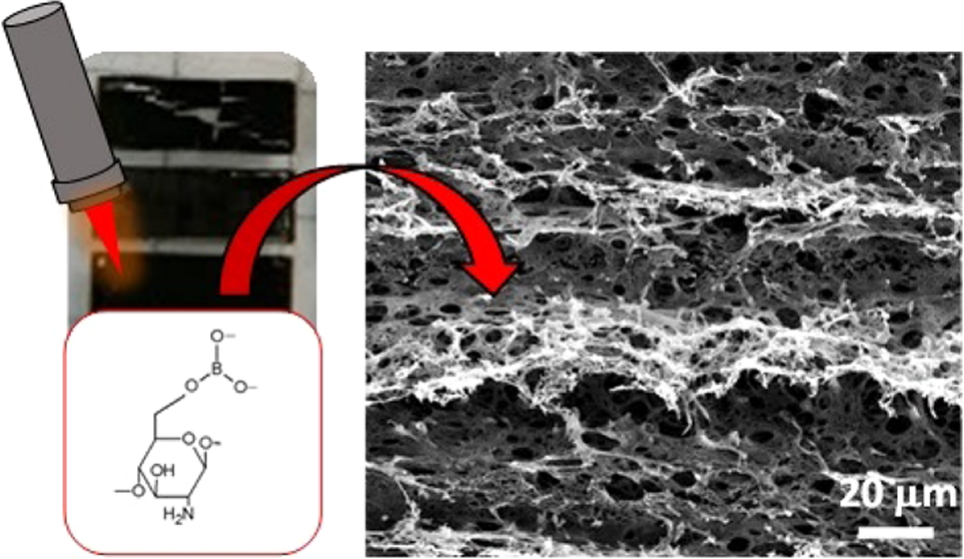

In this study, the laser-induced graphitization process of sustainable chitosan-based formulations was investigated. In particular, optimal lasing conditions were investigated alongside the effect of borax concentration in the chitosan matrix. In all cases, it was found that the obtained formulations were graphitizable with a CO_2_ laser. This process gave rise to the formation of high surface area, porous, and electrically conductive laser-induced graphene (LIG) structures. It was found that borax, as a cross-linker of chitosan, enabled the graphitization process when its content was ≥30 wt % in the chitosan matrix, allowing the formation of an LIG phase with a significant content of graphite-like structures. The graphitization process was investigated by thermogravimetric analysis (TGA), Raman, X-ray photoemission (XPS), and Fourier transform infrared (FTIR) spectroscopies. LIG electrodes obtained from CS/40B formulations displayed a sheet resistance as low as 110 Ω/sq. Electrochemical characterization was performed after a 10 min electrode activation by cycling in 1 M KCl. A heterogeneous electron transfer rate, *k*^0^, of 4 × 10^–3^ cm s^–1^ was determined, indicating rapid electron transfer rates at the electrode surface. These results show promise for the introduction of a new class of sustainable composites for LIG electrochemical sensing platforms.

## Abstract 2

In this study, the laser-induced graphitization process of sustainable chitosan-based formulations was investigated. In particular, optimal lasing conditions were investigated alongside the effect of borax concentration in the chitosan matrix.

## Introduction

Graphene is a material with renowned physical and electrical properties.^1^ Graphene and graphene-like materials are used in areas, such as electronics, electrochemical sensing, strain sensors, energy storage, catalysis, and more.^2−6^ The fabrication of graphene by standard methods, such as chemical vapor deposition, liquid-phase exfoliation, or micromechanical exfoliation, presents challenges relating to achieving a cost-effective and time-effective procedure suitable for commercialization.^7^ Therefore, alternative fabrication methods, such as the use of a laser to reduce graphene oxide, creating graphene-like surface features, have been explored for more than a decade.^8−10^ Recently, direct laser writing protocols have been developed and systematically applied for the conversion of commercial polymers to a high-quality graphene-like material.^11^ Specifically, in 2014, Lin et al. obtained a graphene-like material, termed laser-induced graphene (LIG), by direct laser writing of a commercial Kapton tape (polyimide) with a CO_2_ laser. LIG is a porous, three-dimensional (3D) structure, characterized by a high surface area, resulting from the generation of gases during the lasing process.^12^ The LIG morphology, and hence its surface properties, can be tuned by controlling the laser parameters, such as power, speed, and laser wavelength.^13^ LIG has been shown to be an excellent material for applications, such as electrochemical sensing, energy storage, humidity sensing, and biosensing, among others.^14−20^

Considering the extensive research currently being conducted into LIG-based devices and their wide range of applications, the sustainability of such materials must be considered.^21,22^ In this scenario, it is worth considering alternatives to fossil fuel-derived polymers as precursor materials for LIG production. Although the detailed mechanism of laser-induced polymer graphitization is still not fully clear, it is known that LIG formation results from a combination of photothermal and photochemical processes, highly favored in Kapton by the aromatic structure of polyimide.^23^ In recent years, various natural materials, such as wood, paper, leaves, coal, cloth, and cork, have been used as LIG precursor substrates alternative to polyimide,^15,24−28^ and applications of the resulting “green” LIG structures have ranged from electrochemical sensors to supercapacitors. Natural materials are converted into LIG based on the same principles as polyimide conversion: adsorption of radiation, local increase of temperature, cleavage of C–C, C–O, and C–H bonds, and reorganization and polymerization of the aromatic structures into graphene-like structures.^29^ Recently, attention has also been given to biopolymer materials, in an attempt to widen the range of graphitizable materials. For example, Larrigy et al. have successfully graphitized chitosan films using a multipass laser conversion technique based on a combination of CO_2_ and 405 nm lasers.^30^ The resulting LIG showed a porous morphology with low sheet resistance, below 40 Ω/sq, suitable for electrochemical sensing applications. Huang et al. converted eco-friendly chitosan-based derivatives into LIG with a one-step CO_2_ laser engraving in ambient air.^31^ Low sheet resistances of 12.7 Ω/sq were achieved with chitosan hydrochloride-based sheets; however, these films were too prone to deformation or cracking to be used as devices. Instead, carboxymethyl chitosan-based films, with sheet resistances of 2.2 kΩ/sq, showed suitability for eco-friendly electronics in the form of triboelectric nanogenerators. The introduction of chitosan into the range of natural LIG precursor materials is an important development due to its natural abundance, biocompatibility, biodegradability, nontoxicity, chemical stability, and high reactivity.^32^ However, despite the above attractive properties, chitosan exhibits low chemical stability in wet environments, which limits its application fields.^32^ Sodium tetraborate decahydrate (borax) has been recently investigated as a cross-linking agent to improve chitosan’s mechanical and water barrier properties.^32,33^ Specifically, it has been found that the esterification reaction between borate ions and the hydroxyl moieties of chitosan creates a composite structure denser than pristine chitosan, resulting in increased water and gas barrier properties.^34^ Moreover, the incorporation of boron into cellulose-based materials (cotton, wood, and paper) has been shown to exhibit effective flame-retardant properties, leading to improved limiting oxygen index (LOI) values, reduced peak heat release rate (pHRR), and reduced smoke release.^34,35^ In particular, when mixed to polymer matrices, borax provided a glass-like coating on the fire-exposed surface and promoted the formation of char, which is well known to consist of amorphous carbon as well as graphite-like structures, according to different high-temperature conditions.^36,37^ Coelho et al. and separately Pinheiro et al. have shown that borax-treated chromatography paper can be converted into LIG structures, with electrochemical sensing and microsupercapacitor applications, respectively.^38,39^ The role of borax is elucidated by a comparison with laser-treated raw chromatography paper. While the borax treatment allows the paper to withstand the photothermal degradation process, leading to LIG formation, the raw sample is completely ablated by the laser.

In this work, we introduced borax as a cross-linker of chitosan and investigated the graphitization properties of the resulting formulations by the use of infrared laser sources. Different concentrations of borax were used and it was found that a percentage ≥30% (wt, with respect to CS content) was effective in producing formulations graphitizable by one or two laser passes. The obtained LIG structures displayed high porosity, 3D morphology, and low sheet resistance. The potential use of this LIG material as an electrochemical sensor was investigated. It was found that LIG displayed a high heterogeneous electron transfer rate, comparable to the value found in other green LIG materials, indicating rapid electron transfer rates at the electrode surface. These results show promise for the introduction of a new class of sustainable formulations as LIG feedstock for the development of sustainable electrochemical sensing platforms.

## Experimental Section

### Materials

Medium-molecular-weight chitosan powder was purchased from Sigma-Aldrich. Gluconic acid δ-lactone (GDL) and sodium tetraborate decahydrate (borax) were supplied from Sigma-Aldrich (Italy). All solutions were prepared using deionized Milli-Q water (resistivity 18.2 MΩ.cm).

### CS-Based Film Preparation

Chitosan (CS) powder was dissolved in a D-(+)-gluconic acid δ-lactone (GDL) aqueous solution at room temperature to prepare 20 mg mL^–1^ CS solution. Then, borax aqueous solution was added under stirring. The borax (B) concentration was varied to obtain a final content equal to 20, 30, 40, and 60 wt % with respect to the CS weight. Subsequently, the solution was poured into a glass plate and air-dried to allow solvent removal. The thickness of the obtained films was about 100 μm, measured by a micrometer screw gauge. CS-based films were coded CS/xB, where x represents the weight percentage of borax.

### LIG Fabrication

LIG structures were fabricated by direct laser writing of CS/xB films with a CO_2_ laser engraver (10.6 μm wavelength, 30 W power, HQ-3020B, GuangZhou Amonstar Trade Co., Ltd.). Laser power was varied between 8 and 15% (for higher powers, complete ablation of the material occurred). Laser speed was varied between 10 and 40%. In cases of combinations of low power and high speed, features were overlapped (written once, then again in the same spot, indicated by “×2” in the “Laser settings” column of Table S1) to investigate the effect of multiple laser passes. Conditions were sought, which produced an undamaged, black feature, indicating the formation of LIG.

### Characterization

Surface morphology was analyzed using a Zeiss Supra scanning electron microscope (SEM) equipped with an Oxford X-Max 50 detector operating at an accelerating voltage of 10 kV. Bright-field transmission electron microscopy (TEM) analysis was performed by means of an FEI Tecnai G12 Spirit Twin (LaB6 source) at a 120 kV acceleration voltage. TEM images were collected on an FEI Eagle 4 k charge-coupled device (CCD) camera. Raman investigation was performed with a Renishaw inVia Raman system equipped with a 514 nm argon-ion laser. The focusing of the laser beam onto the sample was obtained through a Leica 20× objective with 0.4 N.A. Spectra were acquired at a laser power of 3 mW and an acquisition time of 10 s. Attenuated total reflection-Fourier transform infrared (ATR-FTIR) analysis was carried out with a portable Bruker α II’s Platinum ATR single-reflection diamond ATR. X-ray photoemission (XPS) spectra were collected by using an Escalab 250Xi (Thermo Fisher Scientific, U.K.) spectrometer, equipped with a monochromatic Al Kα excitation source, electron and ion flood guns for charge neutralization and a 6-channeltron detection system. The photoemission spectra were collected at 20 eV pass energy, and the diameter of the analyzed area was about 1 mm.

Thermal degradation of materials was evaluated by thermogravimetric analysis (TGA), which was carried out under N_2_ flow (flow rate of 40 mL/min) using a TGAQ500-TA Instruments at a heating rate of 10 °C/min in the temperature range from 35 to 1000 °C.

Initial estimations of sheet resistance were made by using a multimeter to measure the resistance across 3 mm × 9 mm features. For CS/B40 and CS/B60 samples, two-terminal current–voltage measurements (±1 V, 20 mV step) were obtained under ambient conditions on transmission line method (TLM) structures using a Wentworth PML 8000 probe station and an Agilent E4980A parameter analyzer. Track resistance values for varying channel lengths were calculated from linear fits of I–V plots. Plots of track resistance versus channel length were used to calculate the sheet resistance (*R*_SH_).

### Electrochemical Measurements

Electrochemical measurements were recorded using a CHI760 bi-potentiostat. A three-electrode setup was used, with Ag/AgCl as the reference electrode and a Pt wire as the counter electrode. LIG was used as the working electrode. An electrode measuring 3 mm × 10 mm was scribed, and the surface was passivated using polyimide tape to reduce the active surface area to approximately 3 mm × 3 mm (see Figure 6a). The measurements were made in a Teflon electrochemical cell with the working electrode at the base. A 10 min electrochemical cleaning cycle was performed using 1 M KCl, in which the potential was varied between −1.6 and 0.8 V at a scan rate of 200 mV/s for 25 cycles. Afterward, 5 mM [Fe(CN)_6_]^3–/4–^ was used for standard electrode characterization. The calculation of the heterogeneous electron transfer (HET) rate constant (*k*^0^) for [Fe(CN)_6_]^3–/4–^ was performed using the method of Nicholson by assuming a transfer coefficient α = 0.5 and using the following diffusion coefficients: *D*_O_ = 7.63 × 10^–6^ cm^2^ s^–1^ and *D*_R_ = 6.32 × 10^–6^ cm^2^s^–1^.^40^

## Results and Discussion

**Figure 1:**
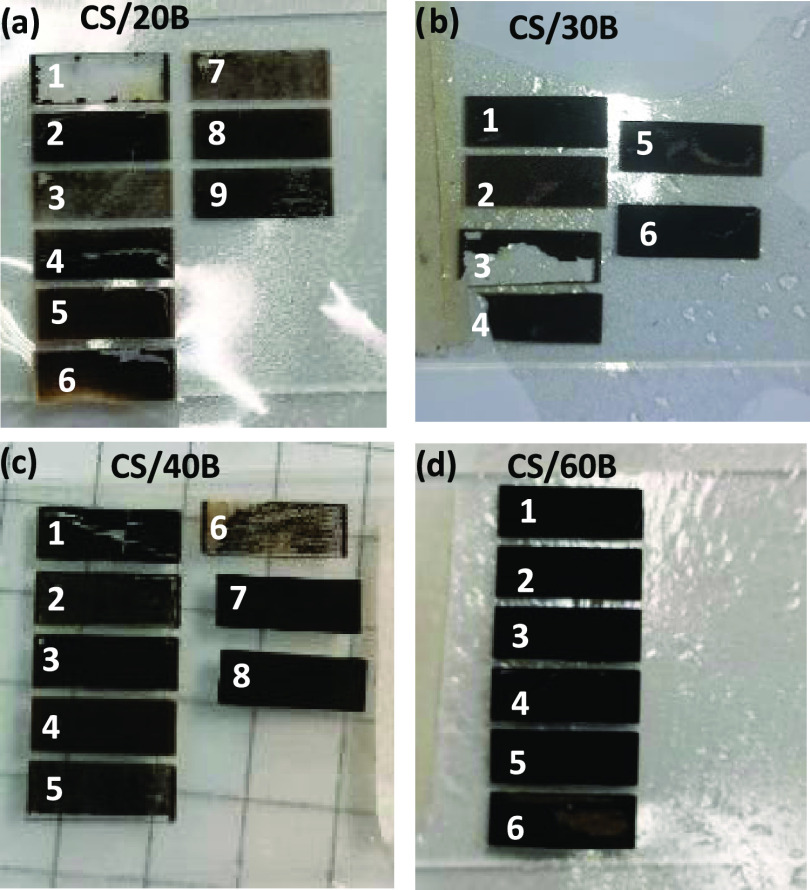
Effect of lasing of (a) CS/20B, (b) CS/30B, (c) CS/40B, and (d) CS/60B films by various laser parameters.

Figure 1 shows photographs of the CS/xB structures obtained at various laser power (LP) and laser speed (LS) values. The initial selection of optimal laser writing parameter optimization was determined by choosing the conditions that produced features with the highest conductivity (see Table S1). Figure 1 shows that the formation of LIG depended on the laser parameters as well as on the content of borax. In detail, Figure 1a shows that for CS/20B formulations, ablation and crack formation occurred for the majority of LP-LS combinations, preventing the formation of fully graphitized and low resistance features. Only one experimental writing condition (LP-LS 10–30 and two overlapped laser passes) led to the formation of LIG features, albeit with high *R*_sh_ ≥ 1 kΩ/sq. An improvement in the graphitization process was observed in Figure 1b, where the borax content was increased to 30 wt %. As shown in Table S1, graphitized structures with *R*_sh_ of about 500 Ω/sq were formed using the combination LP-LS 10–20 and two overlapped laser passes. Figure 1c shows photographs of the features obtained by laser writing of CS/40B formulations, which had the required properties to allow graphitization with a range of laser settings (see details in Table S1), with low *R*_sh_ values reported for LP-LS 10–20 and two overlapped laser passes. Similarly, CS/60B formulations enabled LIG formation over a range of laser settings, as shown in Figure 1d. TLM measurements were performed on both LIG samples, as shown in Figure S1. The *R*_sh_ value for CS/40B-LIG was 110 ± 1, and that for CS/60B-LIG was 341 ± 6 Ω sq^–1^. Overall, these results suggest that increasing the borax content improves the capacity of the material to withstand the high-temperature conditions necessary for the formation of LIG since graphitization of pure CS films with no borax content did not occur under any laser experimental conditions (see Figure S2).

### CS/xB-LIG Characterization

**Figure 2:**
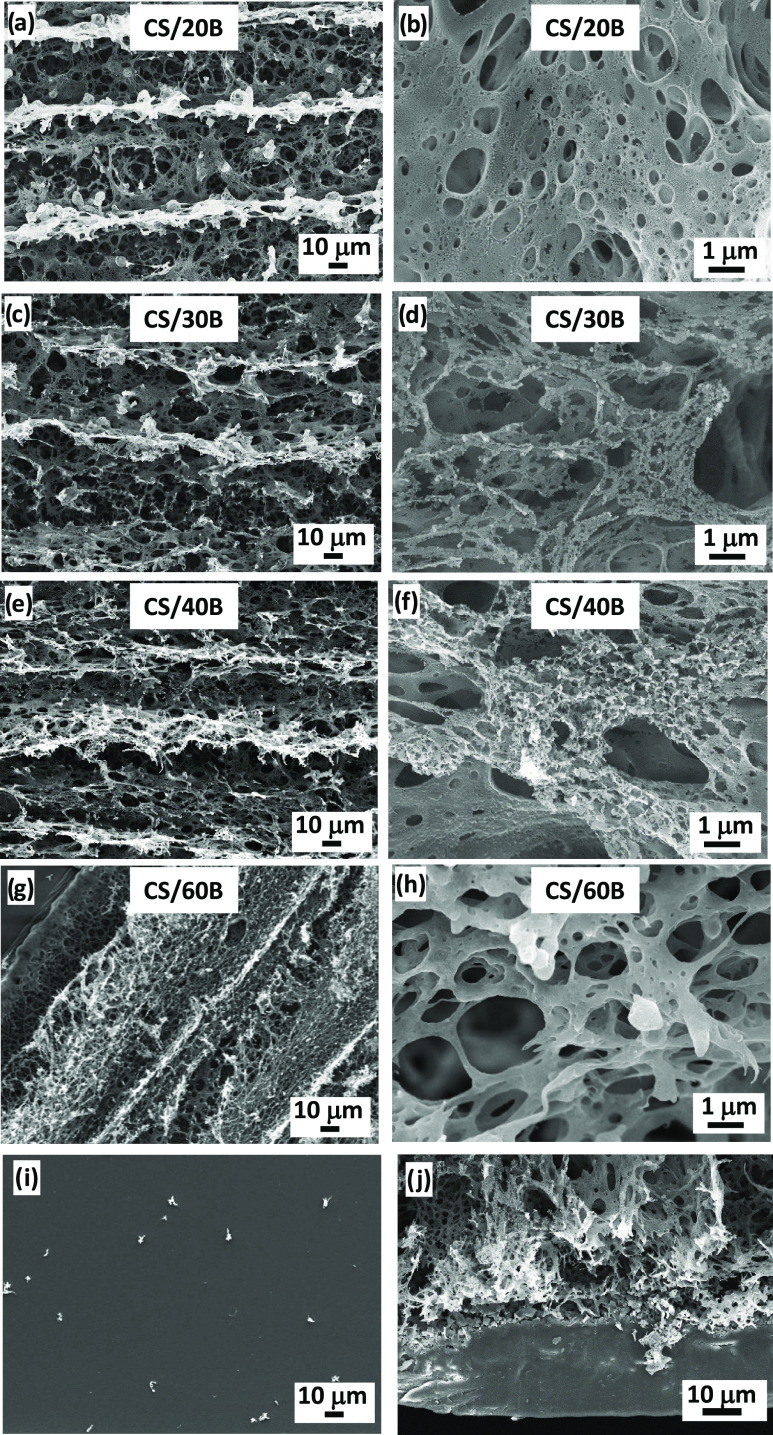
SEM images showing the process of laser graphitization on the CS/xB systems. Low-magnification SEM images of graphitized (a) CS/20B; (c) CS/30B; (e) CS/40B; and (g) CS/60B. High-magnification SEM images of (b) CS/20B; (d) CS/30B; (f) CS/40B; (h) CS/60B; (i) SEM image of pristine CS/40B; and (j) tilted SEM image of graphitized CS/40B.

To investigate the surface morphology, CS/xB-LIG features were examined by scanning electron microscopy (SEM). The SEM images shown in Figure 2a–h confirm that all samples displayed the porous, exploded structure associated with the LIG formation. This is a result of the laser impinging on the biopolymer substrate in the presence of atmospheric oxygen. Similar to what has been reported for laser writing of polyimide and other natural substrates, we speculate that the laser irradiation created a local high-temperature and high-pressure environment, leading to the breaking of the CS’s C–O, C=O, and C–N bonds and to the formation of high-pressure gas pockets that drove the generation of micro and nanopores, along with other structural defects.^14,41−43^ In general, high-magnification SEM images (Figure 2b,d,f,h) showed the formation of a dense and 3D graphitic network, with microholes and tears of different diameters on the LIG surface. Low-magnification images (Figure 2a,c,e,g) displayed the typical peaks and valleys associated with the raster scanning movement of the laser. For comparison, Figure 2i shows the SEM image of a pristine CS/40B film prior to graphitization, which was characterized by a smooth surface. Figure 2j shows a 45° angle tilted SEM image of a graphitized CS/40B sample, showing the LIG formation of about 35 μm thickness.

**Figure 3:**
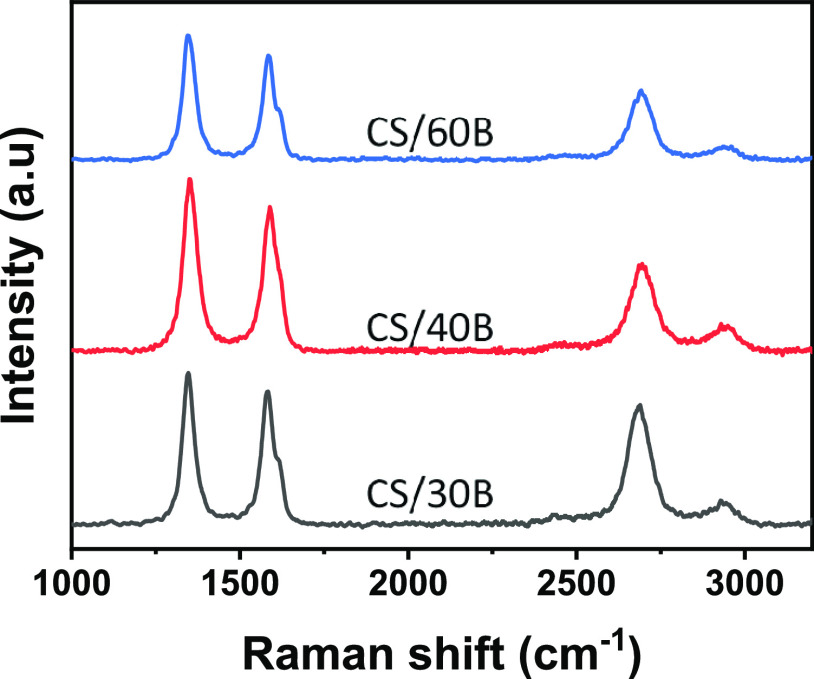
Representative Raman spectra of LIG structures obtained from direct laser writing of CS/xB biopolymer substrates.

Figure 3 shows representative Raman spectra for LIG structures obtained from CS/xB formulations. For CS/20B, Raman investigation confirmed the incomplete graphitization as spectra were either featureless or characterized by broad D, G, and two-dimensional (2D) peaks, highlighting an inhomogeneous distribution of graphene-like structures (see Figure S3).

For borax concentrations of 30 wt % and higher, each Raman spectrum showed well-resolved peaks, characteristic of graphene-like carbon structures: a D mode peak, assigned to a defect-activated radial breathing mode, centered close to 1350 cm^–1^; a G mode peak, related to in-plane bond stretching of pairs of sp^2^ carbon atoms, centered around 1584 cm^–1^; and a 2D mode, the second order overtone of the D peak, centered around 2696 cm^–1^.^44^ A D′ peak was also fitted around 1618 cm^–1^, evident in the asymmetry of the G peak, and associated with the formation of high-quality polycrystalline graphite or creation of defects in natural graphite single crystals.^45,46^ A D + D′ appeared around 2940 cm^–1^ in the higher quality spectra. The formation of D and D + D′ bands is associated with the formation of graphene-like materials with significant defects originating from the intravalley double resonance and a combination mode, respectively.^47^

Peaks were fitted by a single Lorentzian curve. Twenty spectra were collected per LIG sample, with the average fitting results displayed in Table 1. The full width at half-maximum (FWHM) of both G, D, and 2D modes showed little variation across samples (minimum for CS/30B), with all values remaining below 100 cm^–1^. These sharp peaks suggest a high degree of crystallinity, and the FWHM values are in line with high-quality LIG produced on polyimide.^14^ The *I*_D_/*I*_G_ peak ratio was equal to 1 for CS/30B and only slightly increased for borax amounts ≥30 wt %, suggesting a low defect density. This result confirmed the nature of the LIG material, in agreement with the formation of highly ordered nanocrystalline graphitic domains in a disordered carbon matrix.^48^ The relative intensity of the 2D peak also reached a maximum for the CS/30B formulation and then slowly decreased for higher boron concentrations. In parallel, the FWHM of the 2D peak reached a minimum for the CS/30B formulation and then slowly increased for higher boron content. The presence of sharp 2D peaks provided further evidence for a higher degree of ordering, consisting of randomly stacked graphene layers along the *c*-axis,^11^ with the highest order observed for CS/30B and CS/40B structures. The high *I*_2D_/*I*_G_ ratio indicated a low number of graphene layers.^11,48^

**Table 1:** Raman Characteristics of LIG Structures Obtained from Direct Laser Writing of CS/xB Formulation Substrates^a^

sample	FWHM_D_ (cm^–1^)	FWHM_G_ (cm^–1^)	FWHM_2D_ (cm^–1^)	*I*_D/G_	*I*_2D/G_
CS/30B	41	38	69 ± 3	1.0 ± 0.1	0.8 ± 0.1
CS/40B	45 ± 1	39 ± 1	82 ± 2	1.2 ± 0.2	0.5 ± 0.1
CS/60B	49 ± 1	45 ± 1	84 ± 3	1.3 ± 0.1	0.5 ± 0.1

a Error reported is the standard deviation of 20 spectra. No error values were reported for standard deviation less than 0.5 cm^–1^.

The CS/40B formulation was selected as optimal for further investigation and characterization, as it was easily graphitizable, and combined excellent Raman characteristics with the lowest sheet resistance (Figure S1).

### CS/40B-LIG Characterization

**Figure 4:**
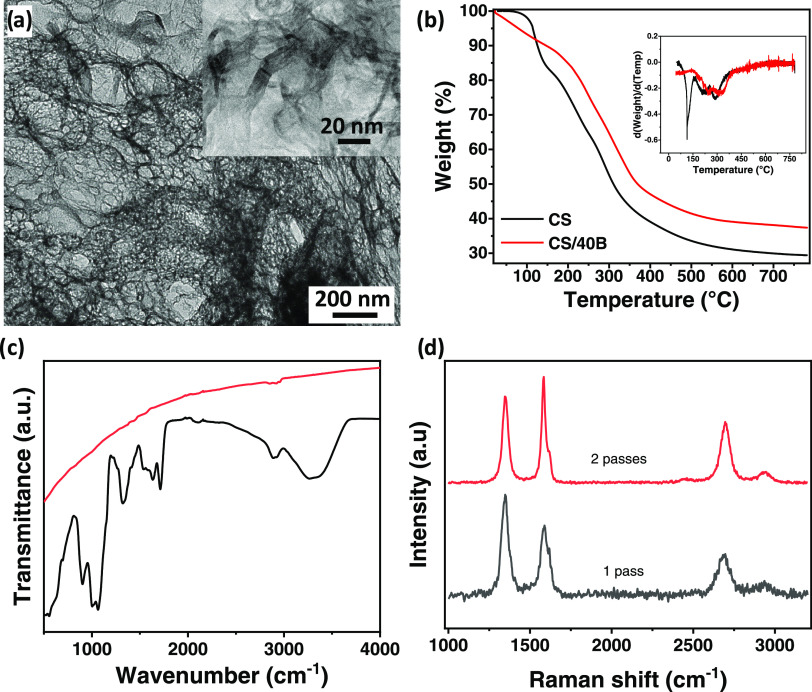
(a) TEM image of the LIG structure obtained from laser writing of CS/40B; (b) TGA of CS and CS/40B films. Inset: DTGA curves; (c) FTIR spectra of pristine CS/40B film (black line) and CS/40B-LIG structure (red line); and (d) Raman spectra after first and second laser pass.

Figure 4a shows a TEM image of a CS/40B-LIG structure displaying crystalline domains, better evidenced in the high-magnification inset, embedded in an amorphous matrix. The high-magnification TEM image (Figure 4a, inset) revealed the formation of multilayer structures, constituted by stacked graphene sheets, as well as nanoscale edges, ripples, and wrinkles, in agreement with the morphology of LIG samples obtained from other polymer and biopolymer matrices.^30^ The calculated interlayer spacing was 3.5 Å (±0.1), which is similar to values determined for LIG on polyimide, wood, and cork.^11,15,24^ Moreover, there is no morphological evidence of a separated borax phase, indicating that borax is incorporated and homogeneously distributed in the LIG structure. Further, TEM images are displayed in Figure S4, alongside details of interlayer spacing calculations.

Figure 4b shows the thermal stability of CS/40B films compared to the CS film, evaluated by TGA. The TGA curves presented different mass loss zones that were better highlighted in the corresponding DTA curves as shown in the inset of Figure 4b. The results show that the CS weight loss took place in two stages. The first one in the region of 60 to 160 °C, with a maximum temperature (*T*d_1_) of 116 °C, was mainly ascribed to water evaporation. The second stage from 160 to 400 °C (two overlapping steps), with a maximum degradation temperature (*T*d_2_) of 288°, was attributed to the pyrolysis of the polysaccharide that presumably started by a random split of the glycosidic bonds, followed by a further decomposition forming predominantly acetic, propionic, and butyric acids.^49^ In the case of CS/40B films, the water loss occurred by a different mechanism compared to bare CS formulations due to the presence of borax, which can bind to water molecules, leading to stronger polymer–water interactions.^50^ Moreover, due to the cross-linking between borax and the chitosan hydroxyl groups, the second stage of degradation showed a *T*d_2_ value of 309 °C, which was 21 °C higher than the *T*d_2_ of the bare CS sample. This temperature shift is probably due to a reduction of the concentration and availability of reactive OH groups in the CS/40B sample. At temperature lower than 400 °C, the anhydrous CS/40B sample converts to aliphatic char, which, in turn, at higher temperatures (≥400 °C) can give rise to cyclization, decarboxylation, decarbonylation, and cross-linking reactions, leading to the formation of aromatic char. It is also important to note that cross-linking of CS with borax increased the residual weight at 800 °C. A residual weight of 29 and 37% was found for CS and CS/40B films, respectively. These results highlight the ability of borax to promote the generation of char components.^32^

The process of LIG formation was analyzed further by spectroscopic characterization. Figure 4c shows ATR-FTIR spectra taken before and after graphitization. The as-fabricated chitosan film showed bands in good agreement with published work on chitosan films.^30,51−54^ In the high wavenumber region of the spectrum (≥2600 cm^–1^), common features were displayed: the band peaking at 3300 cm^–1^ caused by O–H stretching vibrations convoluted with N–H stretching and the band with a peak at 2890 cm^–1^ associated with C–H stretching vibrations. Such stretching vibrations are typical for polysaccharide molecules.^55^ The presence of residual *N*-acetyl groups was confirmed by the presence of bands at 1636 cm^–1^ (C=C stretching of amide I) and 1535 cm^–1^ (N–H bending of amide II), respectively. The absorption peaks at 1064 and 896 cm^–1^ were ascribed to the antisymmetric stretching vibration of C–O–C bridges and glucopyranose ring in the chitosan matrix, suggesting an interaction between the glucopyranose ring in the chitosan matrix and borax.^56^ In contrast, the FTIR spectrum of CS/40B-LIG was featureless, as expected for the conversion to graphitic and amorphous carbon.^15^

A more detailed analysis of the two-step graphitization process was performed by monitoring the change in Raman spectra between the first and second laser pass. The results are displayed in Figure 4d. The progression to a more ordered graphene-like material was clearly seen with the application of a second laser pass as the FWHM of all peaks decreased; the D peak intensity decreased while the G peak and 2D peak intensities increased. Moreover, the *I*_2D_/*I*_G_ increased from 0.4 to 0.5, while the *I*_D_/*I*_G_ ratio decreased from 1.5 to 1.2, indicating the progression to a higher degree of ordering in the sample.

**Figure 5:**
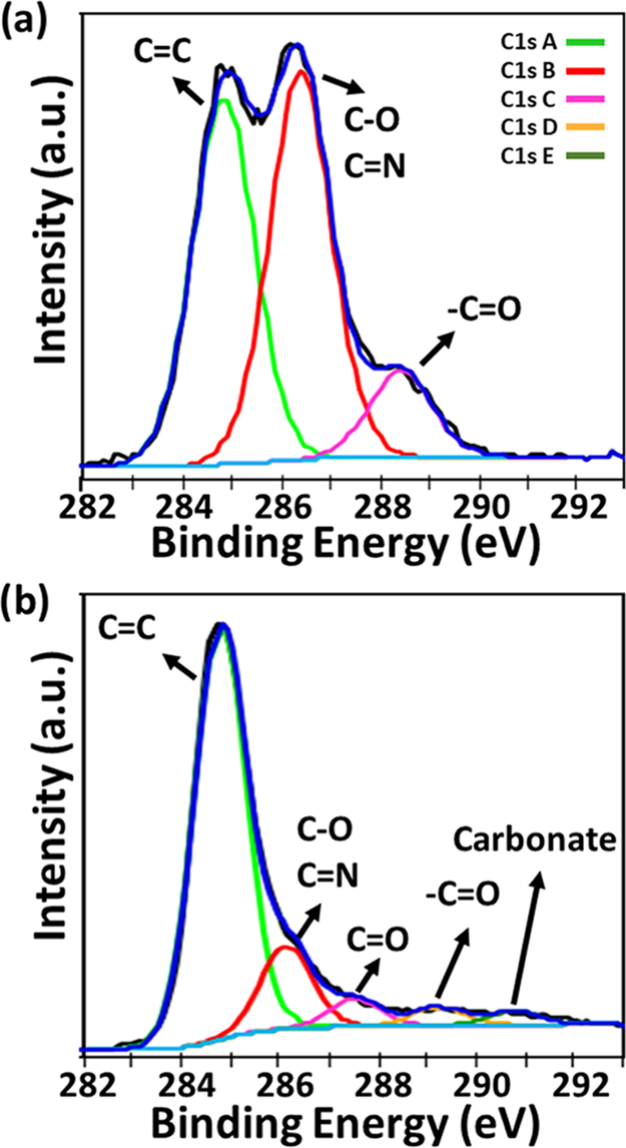
Deconvoluted XPS C 1S spectra of (a) pristine CS/40B film and (b) graphitized CS/40B.

Figure 5 shows the deconvoluted C 1s XPS spectra for the pristine CS/40B formulation and CS/40B-LIG. The graphitized sample was dominated by a large C 1s A peak, representing C=C sp^2^ bonds. Other peaks have been labeled C 1s B (C–C), C (C–OH, −C–O), D (C=O), and E (carbonate). Peak assignments and percentage component concentrations are shown in Table 2. An increase in the percentage of C=C carbon (graphene or graphite) was observed from 26.4% for untreated CS/40B to 52.8% for CS/40B-LIG. The concentration of sp^2^ carbon doubled, and both the ratios of sp^2^ carbon to other bonding forms and to oxygen content more than tripled. Also confirmed was the remaining surface carboxyl and carbonyl groups on the LIG sample, a useful surface feature for electrochemical applications. There was a substantial decrease in the total oxygen concentration in the CS/40B-LIG sample compared to the ungraphitized sample. In contrast, a relative increase in boron (from 2.4 to 7.5%) and sodium (from 3.2 to 4.6%) concentrations was observed in CS/40B-LIG samples compared to the ungraphitized samples (see Figure S5 and Table S3 for full range of XPS data). These data confirmed that borax facilitated the formation of LIG. Also, evidence of the formation of B_2_O_3_ as a result of the graphitization process was found from the XPS data. Overall, the XPS data confirmed that the CS/40B film was converted into LIG with a high sp^2^ carbon content and the presence of surface oxygen groups and that B_2_O_3_ was embedded as an oxide in the LIG network.

**Table 2:** Summary of XPS Data for the CS/40B Pristine Sample and LIG Sample^a^

	atomic percentages (%)		
peak	CS/40B	CS/40B-LIG	assignments
C 1s A	26.4	52.8	C–C sp^2^
C 1s B	28.1	10.5	C–O, C–N
C 1s C	6.2	3.6	C=O
C 1s D		2.4	–C=O
C 1s E		1.8	carbonate
O 1s A	0.5		C–O
O 1s B	31	16.4	O=C
B 1s	2.4	7.5	Na_2_B_4_O_7_ (a), B_2_O_3_ (b)
total C	60.7	71.1	
total O	31.5	16.4	
C 1s A/(B + C + D + E)	0.8	2.9	
C 1s A/O	0.8	3.2	

a For B 1s peak, (a) indicates peak assignment for CS/40B; (b) indicates assignment for CS/40B-LIG.

**Scheme 1:**
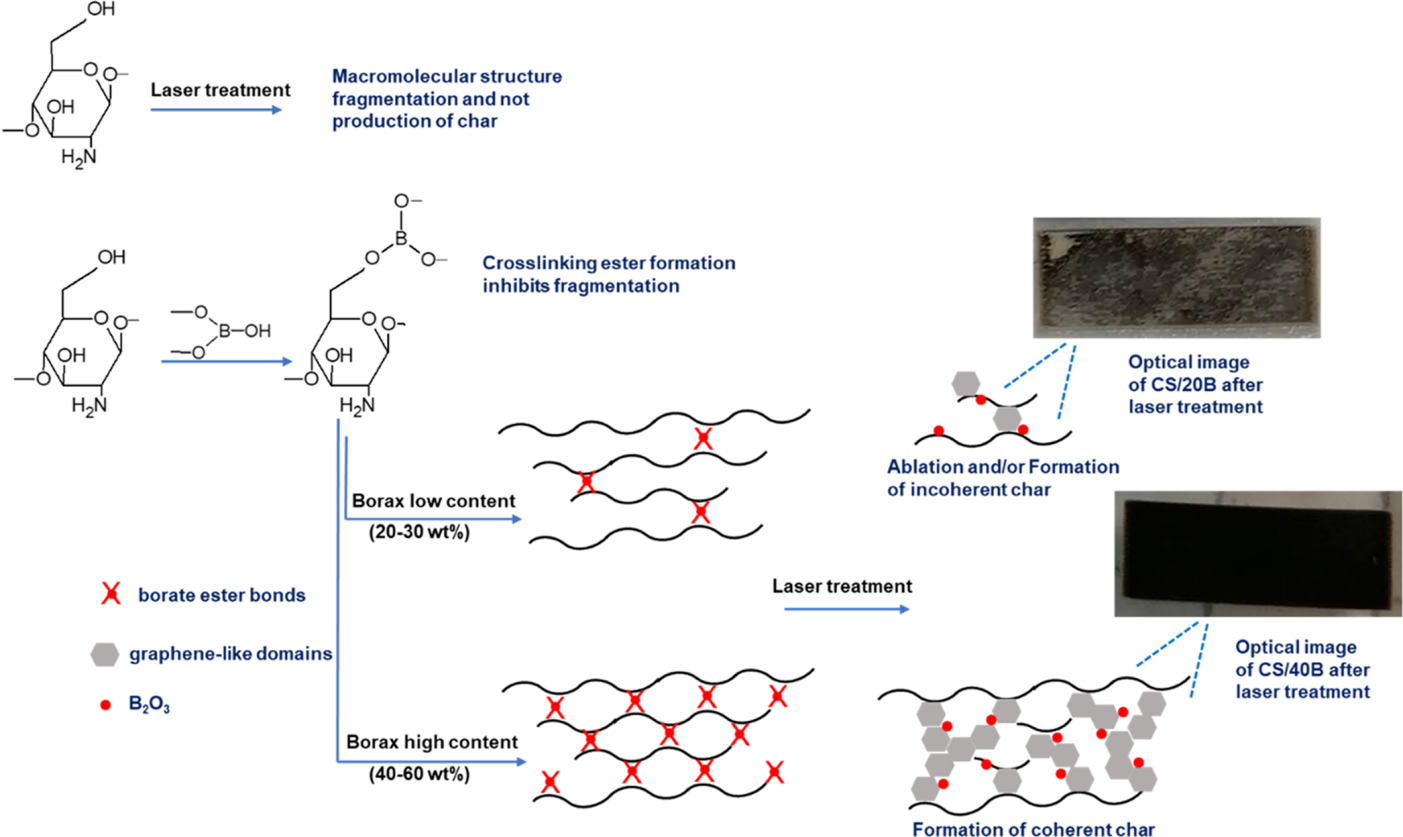
Proposed Mechanism of Action of Borax in LIG Formation

Rationalizing the experimental results, it is possible to speculate that borax, thanks to its intrinsic ability to generate char^34^ and in the case of polysaccharides to cross-link macromolecules,^32^ is the key to allow the formation of graphitizable formulations. In fact, it is well known that borate ions can form covalent bonds with the hydroxyl moieties of CS by forming borate ester bonds, which contribute to densifying the polymeric network.^32,33^ In the absence of B or at B percentage lower than 30 wt %, the direct laser writing of films resulted in CS degradation by polymer weight fragmentation and no formation of aromatic char. The formation of a cross-linked structure inhibits fragmentation of the polymer chain and likely improves char formation by cyclization and condensation.^36^ The proposed graphitization mechanism is shown in Scheme 1.

A comprehensive overview of the characteristics of LIG derived from sustainable sources is provided in Table S2. The Raman characteristics (intensity ratios of D/G and 2D/G peaks) do not vary widely for LIG derived from sustainable precursors. The sheet resistances are generally reported between 10 and 125 Ω/sq, with some materials ranging to kΩ/sq. In this context, the work presented in this paper is more resistive than most reported. Importantly, in terms of application, this material property does not inhibit the performance of electrochemical devices in comparison with those made using chromatography paper, cork, or chitosan.

### Electrochemical Characterization

**Figure 6:**
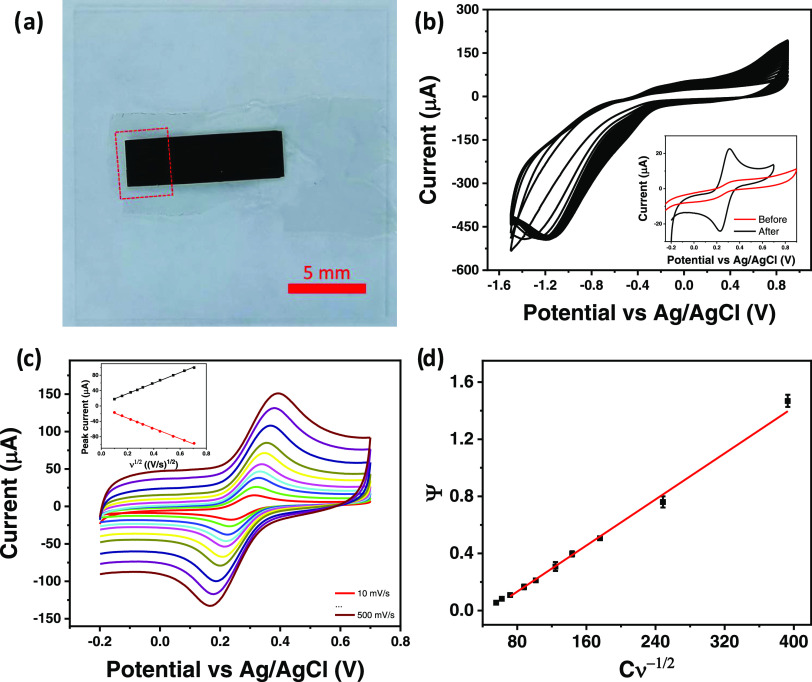
(a) LIG electrode design with exposed area outlined by a dotted red square; (b) cleaning cycles in 1 M KCl with oxygen reduction beginning around −0.5 V and oxygenated group reduction peak appearing around −1.2 V. Inset showing the effect of cleaning cycles on sensing of Fe(CN)_6_^3–/4–^; (c) cyclic voltammetry (CV) scans at varying scan rates for 5 mM Fe(CN)_6_^3–/4–^ in 1 M KCl. Inset shows peak oxidation and reduction current with the square root of scan rate; (d) *k*^0^ calculation by the Nicholson method.

An electrochemical characterization of CS/40B-LIG was performed to assess its capability as an electrode material for future electrochemical applications. Characterization was performed with a three-electrode electrochemical setup, whereby LIG was used as the working electrode. [Fe(CN)_6_]^3–/4–^, a standard redox probe, which undergoes a reversible one-electron reaction, was used for this investigation. Cyclic voltammetry (CV) was employed to investigate the electron transfer kinetics of the CS/40B-LIG electrode. Figure 6a shows the electrode design used, with the dotted line, indicating the area exposed to the analyte. The electrochemical cleaning of the electrode surface is shown in Figure 6b. The result of cycling in 1 M KCl for 10 min was the reduction of oxygen and oxygenated groups at −0.4 and −1.2 V, respectively.^57^

The effect of this electrochemical cleaning is seen in the inset of Figure 6b. The oxidation and reduction peak currents of [Fe(CN)_6_]^3–/4–^ increased in magnitude, the peaks became sharper, and a reduced peak separation was observed. Figure 6c shows the cyclic voltammetry curves of Fe(CN)_6_^3–/4–^ measured between 10 and 100 mV/s scan rates. Fast electron transfer rates were observed, with Δ*E*_P_ values below 80 mV at 10 mV/s and below 200 mV up to scan rates of 500 mV/s. The inset of Figure 6c shows the linear plots of peak current (oxidation current and reduction current) with the square root of the scan rate, in line with the Randles–Ševčík equation and indicating a semi-infinite linear diffusion regime. Monitoring the values of Δ*E*_P_ with changing scan rate allowed for the determination of the hetero electron transfer rate (*k*^0^) by the Nicholas method. The value calculated for this material was 4 × 10^–3^ cm s^–1^. A comparison with literature values for [Fe(CN)_6_]^3–/4^, including LIG on polyimide as a benchmark, is provided in Table 3. The *k*^0^ value presented in this work is an order of magnitude larger than values seen for LIG on paper substrates and much improved from electrochemical results previously reported for LIG on a chitosan-based biofilm. In (59), the electrochemical sensor was water-soluble, leading to a breakdown with time. The improvement of the water barrier properties of the chitosan–borax films presented in this paper allows for more stable devices. The rapid electron transfer kinetics presented here mean that this material is suitable for further use as an electrochemical sensing platform.

**Table 3:** Comparison of Electron Transfer Properties for Ferrocyanide with Literature Values for LIG on Polyimide and Natural Precursors^a^

material	laser	Δ*E*_P_ (mV)	*k*_app_^0^ (cm s^–1^)	refs
polyimide	450 nm	61	1.26 × 10^–1^	(14)
polyimide	405 nm	86	1.3 × 10^–2^	(48)
filter paper	10.6 μm		7.8 × 10^–4^	(58)
office paper	10.6 μm	≥700	4.08 × 10^–4^	(39)
chromatography paper	10.6 μm	370	6.85 × 10^–4^	(39)
chitosan	10.6 and 405 nm	290		(59)
cork	450 nm	90	9 × 10^–3^	(60)
chitosan–borax	10.6 μm	110	4 × 10^–3^	this work

a Δ*E*_P_ for ν between 50–150 mV s^–1^ (100 mV s^–1^ for this work).

These results present an advanced material from renewable sources. Green LIG sensors are suitable for a variety of Internet of Things applications. The surface qualities of LIG allow for facile modification of the surface, making this an ideal platform for a green-biosensing platform.

## Conclusions

The successful conversion of CS/xB formulations into high-quality LIG has been presented. It was found that 40% wt. borax (with respect to CS weight) was the optimal content by monitoring the electrical and Raman parameters of LIG. Borax content in the formulations’ composition was found to play an important role in the formation of LIG by acting as a cross-linking agent for chitosan and due to its flame-retardant properties, which facilitated the high-temperature conditions necessary for LIG formation. The derived CS/40B-LIG displayed excellent properties as an electrochemical sensing platform. The *k*^0^ value determined for [Fe(CN)_6_], 4 × 10^–3^ cm s^–1^ is higher than that calculated for most other green LIG sensors and is higher than previous works on chitosan-based LIG. This showcases the potential for this material in future electrochemical applications. These CS-based films offer an important, environmentally friendly route to creating LIG materials, an alternative to fossil fuel-derived synthetic polymers.

## Data Availability

The data presented in this article are available from the author upon reasonable request.

## Supporting Information Available

Additional electrical data for optimizing laser parameters; chitosan sheet in the absence of borax; Raman spectroscopy of CS/20B; table of comparison with literature for LIG from natural precursors; additional TEM images and lattice spacing calculations; XPS O 1s, B 1s and Na 1s spectra for CS/40B-LIG; HET rate calculations (PDF)
