# Supplementary material for: Direct Laser Writing of Chitosan–Borax Composites: Toward Sustainable Electrochemical Sensors
Source: ACS Sustain Chem Eng. 2023 Sep 1;11(37):13574–83. doi: 10.1021/acssuschemeng.3c02708 (PMC10521144; doi:10.1021/acssuschemeng.3c02708)
Supplement: Supplementary file 1 — sc3c02708_si_001.pdf [file sc3c02708_si_001.pdf]

Supporting Information Direct Laser Writing of Chitosan-Borax Composites: Towards Sustainable Electrochemical Sensors

Eoghan Vaughan,[a†] Chiara Santillo,[b†] Alessandra Imbrogno,[a] Gennaro Gentile,[c] Aidan J. Quinn,[a] Saulius Kaciulis,[d] Marino Lavorgna,[b*] Daniela Iacopino[a]*

[a]Tyndall National Institute, University College Cork, Lee Maltings Complex, Dyke Parade, Cork, Ireland

[b]Institute for Polymers, Composites and Biomaterials, National Research Council of Italy, P.le E. Fermi 1, 80055 Portici, Italy.

[c]Institute for Polymers Composites and Biomaterials, National Research Council of Italy, Via Campi Flegrei 34, 80078 Pozzuoli, Italy.

[d]Institute for the Study of Nanostructured Materials, National Research Council, 00015, Monterotondo Staz., RM, Italy

† These authors contributed equally

* Co-corresponding authors: Daniela Iacopino and Marino Lavorgna

E-mail addresses: Daniela.iacopino@tyndall.ie and mlavorgna@unina.it

CS/20B Electrical Data

Table S1 Laser settings applied to CS/20B sample and corresponding sheet resistance measurements.

CS/20B	Settings (LP-SP)	R_{sh} (Ω/sq)	CS/B30	Settings (LP-SP)	R_{sh} (Ω/sq)	CS/B40	Settings (LP-SP)	R_{sh} (Ω/sq)	CS/B60	Settings (LP-SP)	R_{sh} (Ω/sq)
1	10-20 (x2)	N/A	1	10-20 (x2)	470	1	10-10	N/A	1	10-20 (x2)	300
2	10-30 (x2)	N/A	2	11-20 (x2)	N/A	2	12-12	4.3 k	2	10-20	1 k
3	10-40 (x2)	N/A	3	11-25 (x2)	N/A	3	12-15	1.3 k	3	12-15	0.8 k
4	10-40 (x3)	N/A	4	12-30 (x2)	500	4	12-20	4 k	4	12-12	0.7 k
5	12-40 (x2)	N/A				5	13-13	5.6 k	5	10-12	500
6	11-40 (x2)	N/A				6	15-15	N/A	6	12-30 (x2)	N/A
7	14-30	N/A				7	10-20 (x2)	100			
8	8-15	N/A				8	10-20 (x3)	N/A			
9	8-20	N/A									
10	9-20	N/A									
11	15-50	N/A									
12	10-30 (x2)	1.3 k									
13	10-25	N/A									

For these TLM structures, 2 x 20 mm (10 squares) LIG features were scribed, with measurements taken at track lengths L1 = 4 mm, L2= 8 mm, L3 = 12 mm, L4 = 16 mm, L5 = 20 mm, beginning with the largest separation to avoid damaging the track between terminals for proceeding measurements. A square (S) is defined simply as a feature with length equal to width, and the number of squares is the track length divided by the width. The track resistance (Ri) between two consecutive pads could then be related to the number of squares via equation S1:

$$Ri = Rc + (Rs)(S) \quad \text{(S1)}$$

where Rc is the contact resistance, Rs is the sheet resistance of the sample. Values for Rs were thus obtained from linear regression of Ri versus S, as shown in Figure S1. The value for CS/40B-LIG was 110 ± 1 Ω sq^{-1}, and 341 ± 6 Ω sq^{-1} for CS/60B-LIG. TLM measurements were not taken for CS/30B, as the material could not support the required features.

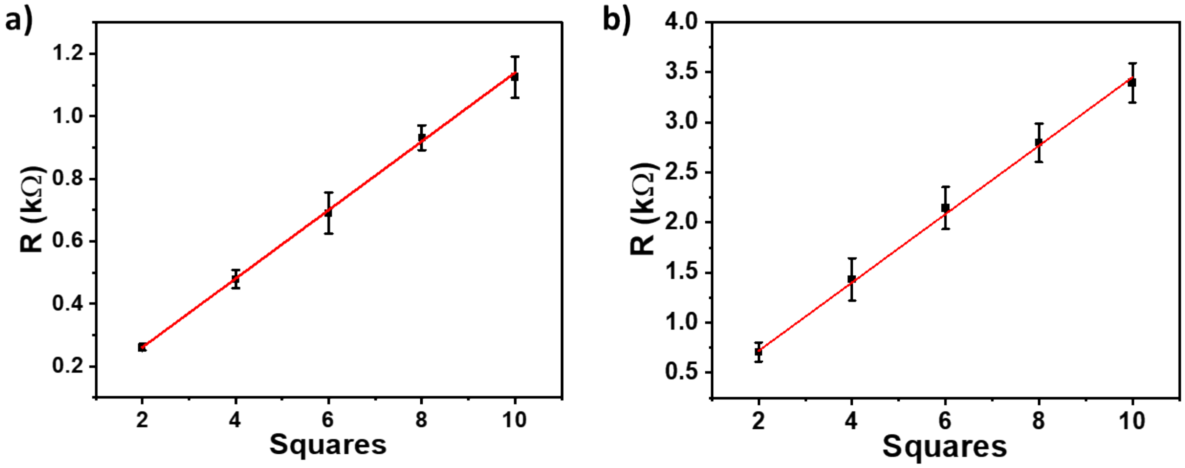

Figure S1 TLM measurements for **a)** CS/40B-LIG and **b)** CS/60B-LIG. Both LIG formations were at laser power 10% and speed 20%, with two overlapped laser passes.

Chitosan sample

Figure S2 shows a photograph of a CS material without the inclusion of Borax. A column of features is visible on the left, which were created by the CO_2 laser, and represent unsuccessful attempts at graphitising the material. No evidence of the formation of char (black) features was

seen across a range of laser settings, and attempts at using multiple laser passes resulted in complete ablation of the material.

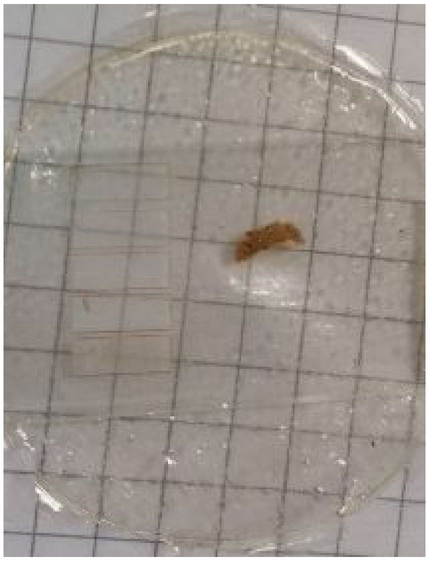

Figure S2 Bare chitosan sample showing unsuccessful graphitisation.

Raman spectroscopy

For CS/20B, Raman investigation confirmed the incomplete graphitization. Of the 17 spectra that were recorded: 10 showed no peaks; 3 showed weak and broad D and G peaks; 4 spectra showed broad D, G and 2D peaks. Examples of shown in Figure S1.

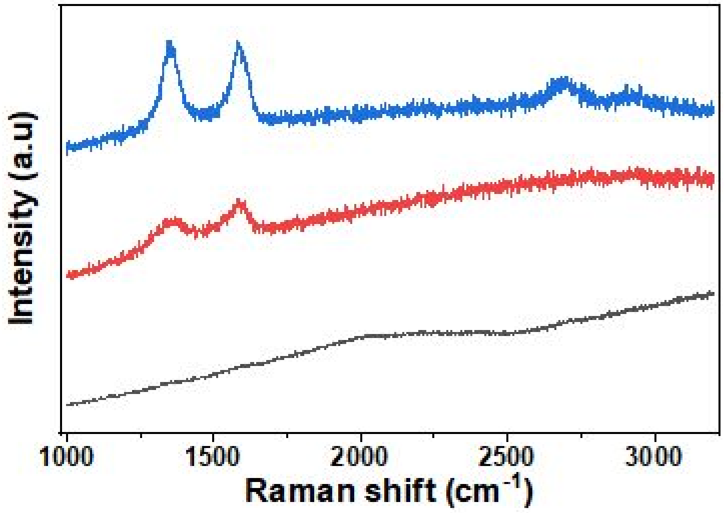

Figure S3 Three types of Raman spectra recorded for CS/20B sample: featureless (black; 10/17); weak, broad D and G peaks (red; 3/17); weak, broad D, G and 2D peaks (blue; 4/17).

Table S2 Comparison of CS/40B-LIG material characteristics with literature data.

Material	Laser	Raman (D/G)	Raman (2D/G)	R_{sh} (Ω/sq)	Application	Ref
Paper	UV (355 nm)			125	Humidity and temp. sensor	[1]
Wood	IR (10.6 μm)	0.8	0.5	10	Supercapacitors, water splitting electrodes	[2]
Watercolor paper	IR	1		40	-	[3]
Chromatography paper	IR	0.5		32	Strain sensor	[4]
Chromatography paper	IR	1.28	0.62	56	Electrochemical sensor	[5]
Chromatography paper	IR	>1	<1	30	Supercapacitor	[6]
Cellulose nanofibers	IR			16 k	-	[7]
Cork	UV	0.41	0.37	75	Pressure sensor	[8]
Cork	Visible (450 nm)	1	0.37	46	Electrochemical sensor	[9]

Chitosan	Visible (405 nm) & IR	~1		40	Electrochemical sensor	[10]
Carboxymethyl chitosan	IR	1.6	0.4	2.2 k	Triboelectric nanogenerators	[11]
Chitosan-borax composite	IR (10.6 μm)	1.2	0.5	110	Electrochemical sensor	This work

Lattice Spacing Calculation

Lattice spacing was determined by examination of TEM images. Multilayer regions were identified as shown in Figure S1, and their spacing profile plotted. Inter-peak separations were determined and their means calculated.

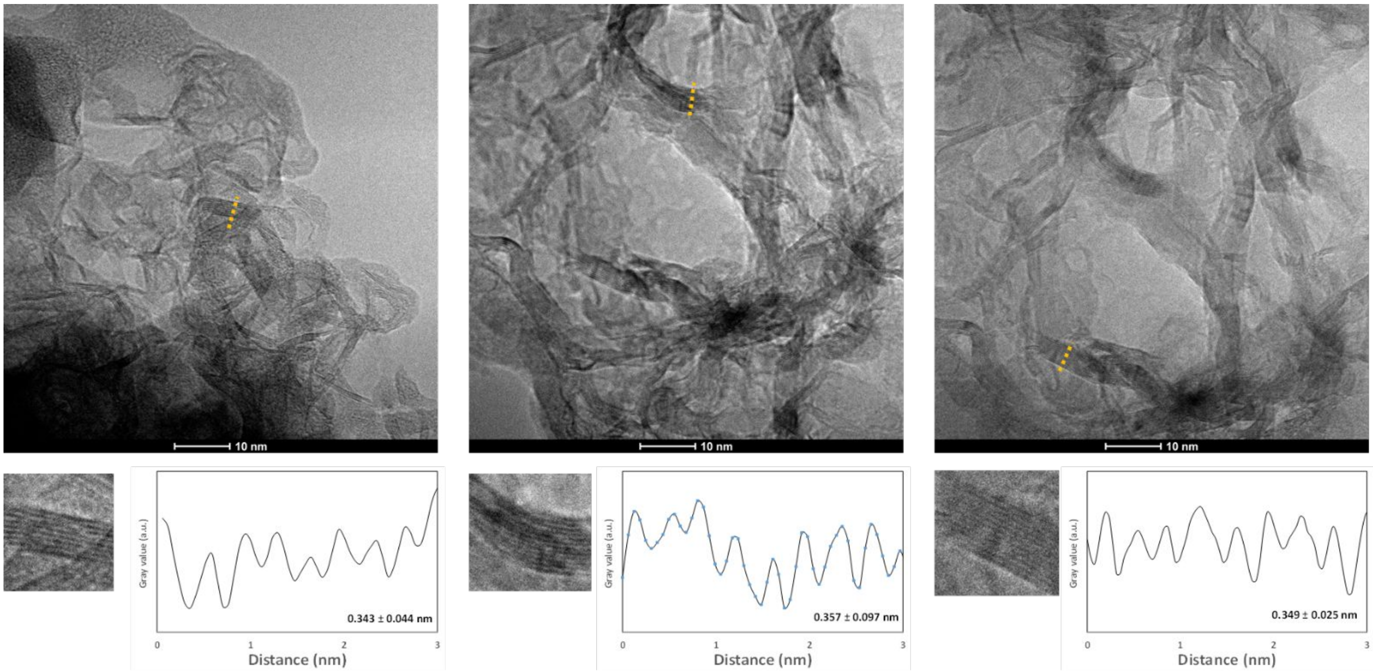

Figure S4 TEM images from which lattice spacing was calculated. Highlighted multilayer regions are shown alongside the spacing profile plots.

XPS Data

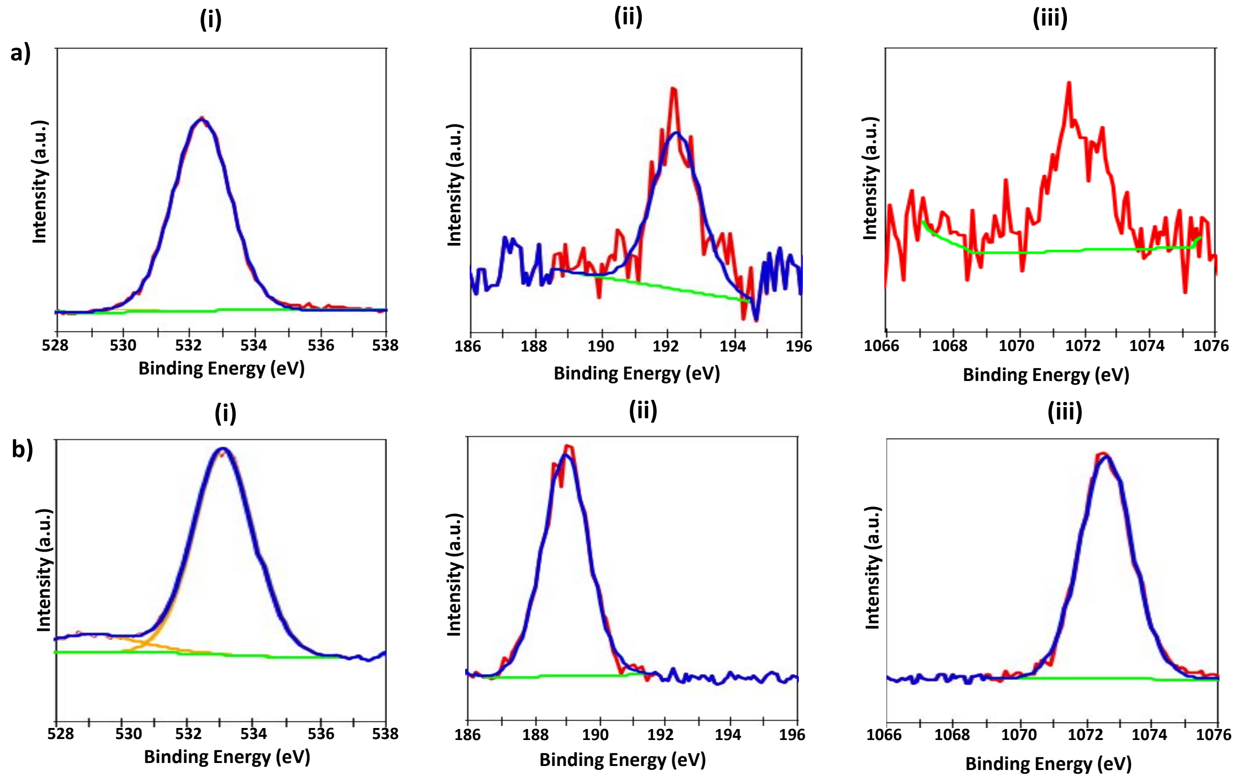

Figure S5 XPS **(i)** O1s, **(ii)** B1s, and **(iii)** Na1s spectra of **a)** pristine CS/40B and **b)** CS/40B-LIG

Table S3 Full range of XPS data for **(a)** CS/40B and **(b)** CS/40B-LIG.

(a)	Peak	BE (eV)	FWHM (eV)	Atomic %	State
	B1s	192.3	1.73	2.4	$Na_2B_4O_7$
	C1s - A	284.8	1.48	26.4	C-C sp^2
	C1s - B	286.4	1.48	28.1	C-O, C=N
	C1s - C	288.4	1.48	6.2	-C=O
	N1s - A	399.8	1.93	1.7	C-NH-, -NH_2
	N1s - B	402.0	1.93	1.5	NH^{4+}

Na1s	1071.5	0.67	1.4	Na^{1+}
O1s - A	530.2	2.06	0.5	oxides
O1s - B	532.3	2.06	31.0	C=O,OH^-
Si2p	102.7	0.31	0.9	SiO_2

(b)

Peak	BE (eV)	FWHM (eV)	Atomic %	State
B1s	193.1	1.65	7.5	B_2O_3
C1s - A	284.8	1.23	52.8	C-C sp^2
C1s - B	286.1	1.23	10.5	C-O, C=N
C1s - C	287.5	1.23	3.6	C=O
C1s - D	289.2	1.23	2.4	-C=O
C1s - E	290.8	1.23	1.8	carbonate
N1s	399.9	0.26	0.3	O=C-NH-, -NH_2
Na1s	1072.6	1.87	4.6	Na^{1+}
O1s	532.9	2.14	16.4	C=O, B_2O_3

Heterogeneous Electron Transfer (HET) coefficient rate calculations

HET rates (k^0) were calculated using the Nicholson method.[12] The following equation was used to relate k^0 to the dimensionless kinetic parameter Ψ:

$$\Psi = k^0\left(\frac{D_O}{D_R}\right)^{\frac{\alpha}{2}}\sqrt{\frac{RT}{n\pi F D_O \nu}} \qquad \text{(S2)}$$

where D_O and D_R are the diffusion coefficients of the oxidised/reduced forms of the analyte, α is the charge transfer coefficient, *n* is the number of electrons involved in the electron transfer reaction, ν is the scan rate (Vs^{-1}), F is the Faraday constant, R is the universal gas constant and T is the absolute temperature. Equation (S2) can be simplified to:

$$\Psi = k^0 C\nu^{-1/2} \qquad \text{(S3)}$$

where C is a constant characteristic of the given analyte under investigation. The following equation was used to relate the peak separation (ΔE_P) to k^0 [13]

$$\Psi = \frac{(-0.6288 + 0.0021X)}{(1 - 0.017X)} \qquad \text{(S4)}$$

where X is equal to ΔE_P (mV) multiplied by the number of electrons involved in the reaction.

References

[1] Kulyk, B., Silva, B. F. R., Carvalho, A. F., Barbosa, P., Girão, A. V., Deuermeier, J., Fernandes, A. J. S., Figueiredo, F. M. L., Fortunato, E., Costa, F. M., Laser-Induced Graphene from Paper by Ultraviolet Irradiation: Humidity and Temperature Sensors. Adv. Mater. Technol. 2022, 7, 2101311. https://doi.org/10.1002/admt.202101311

[2] Ye, R., Chyan, Y., Zhang, J., Li, Y., Han, X., Kittrell, C., Tour, J. M., Adv. Mater. 2017, 29, 1702211. https://doi.org/10.1002/adma.201702211

[3] Yieu Chyan, Joseph Cohen, Winston Wang, Chenhao Zhang, and James M. Tour. ACS Applied Nano Materials 2019 2 (5), 3007-3011. DOI: 10.1021/acsanm.9b00391

[4] Bohdan Kulyk, Beatriz F. R. Silva, Alexandre F. Carvalho, Sara Silvestre, António J. S. Fernandes, Rodrigo Martins, Elvira Fortunato, and Florinda M. Costa. ACS Applied Materials & Interfaces 2021 13 (8), 10210-10221. DOI: 10.1021/acsami.0c20270

[5] Pinheiro, T., Silvestre, S., Coelho, J., Marques, A. C., Martins, R., Sales, M. G. F., Fortunato, E., Laser-Induced Graphene on Paper toward Efficient Fabrication of Flexible, Planar Electrodes for Electrochemical Sensing. Adv. Mater. Interfaces 2021, 8, 2101502. https://doi.org/10.1002/admi.202101502

[6] Coelho, J., Correia, R.F., Silvestre, S. et al. Paper-based laser-induced graphene for sustainable and flexible microsupercapacitor applications. Microchim Acta 190, 40 (2023). https://doi.org/10.1007/s00604-022-05610-0

[7] Sanghee Lee and Sangmin Jeon; ACS Sustainable Chemistry & Engineering 2019 7 (2), 2270-2275. DOI: 10.1021/acssuschemeng.8b04955

[8] Carvalho, A. F., Fernandes, A. J. S., Martins, R., Fortunato, E., Costa, F. M., Laser-Induced Graphene Piezoresistive Sensors Synthesized Directly on Cork Insoles for Gait Analysis. Adv. Mater. Technol. 2020, 5, 2000630. https://doi.org/10.1002/admt.202000630

[9] Vaughan, E., Santillo, C., Setti, M., Larrigy, C., Quinn, A.J., Gentile, G., Lavorgna, M. and Iacopino, D. (2023), Sustainable Laser-Induced Graphene Electrochemical Sensors from Natural Cork for Sensitive Tyrosine Detection. Adv. Sensor Res. 2300026. https://doi.org/10.1002/adsr.202300026

[10] Larrigy, C., Burke, M., Imbrogno, A., Vaughan, E., Santillo, C., Lavorgna, M., Sygellou, L., Paterakis, G., Galiotis, C., Iacopino, D., Quinn, A. J., Porous 3D Graphene from

Sustainable Materials: Laser Graphitization of Chitosan. Adv. Mater. Technol. 2023, 8, 2201228. https://doi.org/10.1002/admt.202201228

[11] Qian-Ming Huang, Huiru Yang, Shaogang Wang, Xu Liu, Chunjian Tan, Anxin Luo, Siyuan Xu, Guoqi Zhang, and Huaiyu Ye; ACS Applied Nano Materials 2023 6 (12), 10453-10465. DOI: 10.1021/acsanm.3c01408

[12] Nicholson, R.S., *Theory and Applicatino of Cyclic Voltammetry for Measurement of Electrode Reaction Kinetics.* Analytical Chemistry, 1965. **37**(11): p. 1351-1355

[13] Lavagnini, I.; Antiochia, R.; Magno, F. An extended method for the practical evaluation of the standard rate constant from cyclic voltammetric data. *Electroanalysis,* **2004**, 16, 6, 505-506.
